# The Hospital. Nursing Mirror

**Published:** 1898-08-13

**Authors:** 


### "The Hospital" Nursing Mirror.

Being the Nursing Section of "The Hospital."

[Contributions for this Section of "The Hospital" should be addressed to the Editor, The Hospital, 28 & 29, Southampton Street, Strand, London, W.C., and should have the word "Nursing" plainly written in left-hand top corner of the envelope.]

### News from the Nursing World.

### THE RED CROSS IN THE FIELD.

The various journals are now full of communications from their respective war correspondents. They tell of battles with their harvests of slain and wounded, of inadequate ambulance and surgical service, and of the welcome aid given by the Red Cross Society. It is the unexpected that comes to pass, and this is doubly true in war. The American Medical Department was not prepared for the severe engagements that took place at Santiago de Cuba, and at first refused the help of the Red Cross Society's hospital ship lying at Siboney with supplies for the Cuban reconcendrados. But they were glad to invoke its aid before the end, for the sick and wounded came thronging down over the eight miles that lay between Santiago and Siboney in ammunition waggons, on improvised crutches, and on foot. The hospital was full, cots were needed, supplies were short, nurses were wanted, and all these the Red Cross ship sent. There will be many heartburnings amongst the American people when they read of the soldiers' sufferings, for any number of trained nurses had volunteered their help without remuneration, and the nursing societies were ready to guarantee skilled nurses by the thousand. "Brother Jonathan" has doubtless learned his lesson well, but the fees of that most excellent schoolmaster, experience, have been in this instance indeed heavy.

### THE BRABAZON HOME OF COMFORT.

The Brabazon Home of Comfort at Reigate quietly does a great deal of good at very little cost. Everyone knows how much may be done for delicate girls by judicious and careful nursing at such times as their health fails either through illness or through overwork; and many lives are saved and years of suffering avoided. The Home of Comfort is a veritable haven of refuge to such as are admitted. Some are nursed back to life and self-support, others to their entering on their final rest. They are tended physically and mentally, and their lives made bearable and, not infrequently, happy whilst at the Home. Each patient costs £25 a year, to which sum most of the inmates contribute a certain proportion. The total expenses of the home average £800 a year, and subscriptions to the amount of £200 are necessary. The anniversary of the opening of rhe Brabazon Home was held last month, when a large number of friends visited it, and a sale of the patients work realised £11. Any of our readers who may feel inclined to help it may, by applying to the secretary, receive a full account of it, and of the lines on which it is carried on. Gifts of money are, of course, most welcome, but presents of clothing and provisions are also very useful. Just now new kitchens are to be built by the doctor's orders, and this outlay will tax very heavily the slender reserve fund. Two points should be noted—only girls of good character are received; and they are kept at the home as long as they need it, provided, of course, that their cases are suitable.

### THE VICTORIA HOME, LANCASTER.

The Hon. Secretary of the Lancaster District Nursing Society announces that the fund for establishing a home for the District Nurses of Lancaster has reached £700. A house—No. 33, Regent Street—has been rented from August 13th, and the furnishing will be begun at once. Gifts of furniture and other household plenishings are invited. When a favourable opportunity presents itself it is intended to secure a house in a more central position.

### NURSES' SALARIES IN NEW SOUTH WALES.

For a long time reforms in the admistration of the Coast Hospital, Little Bay, Sydney, have been badly needed, and the attention of the Government has been drawn to the matter on more than one occasion, and now the Public Service Board has recently made a move in the right direction by revising the scale of salaries paid to nurses on the staff. They will for the future be as follows: The head nurse is to receive £55, the senior nurses £50 each, the nurses £40, the junior nurses £35 (the maximum), and the probationers £25. Temporary nurses also get £25 a year. The permanent staff is recruited from the temporary nurses who have been employed for three months. After appointment they serve as probationers for nine months.

### GOSPORT NURSING SOCIETY.

The Victoria Nursing Society, Gosport, has recently presented its first annual report to its supporters. Two nurses have been at work since December last, and the balance-sheet is a very satisfactory one. The society was founded in memory of the Diamond Jubilee, and the Princess Henry of Battenberg is the president. Donations to the amount of £2,000 have been invested, and after all expenses have been paid the treasurer has £27 in hand; and the members of the Gosport Cycling and Athletic Club have handed over to the committee £32, being two-thirds of the profits of some sports held in Gosport Park.

### THE MELBOURNE NURSING SOCIETY.

A series of meetings are now being held in order to prepare for a bazaar that is to take place in Melbourne Town Hall, on September 2nd and 3rd, in aid of the Melbourne District Nursing Society. Eleven stalls and a Christmas tree have been allotted to various ladies, and the following prizes have been offered for competition: Lady Clarke and Mrs. George Chirnside one of £5 5s. for a silk blouse; Lady Sargood, £2 2s. for the best dressed doll; and different ladies give prizes, valuing £1 1s. each, for hand-painted picture frame, child's frock, a lamp shade, &c., respectively. Sir John Madden has promised to open the bazaar.

### A QUEEN'S NURSE FOR NEWRY.

Mr. Henry Thomson, J.P., of Ballyedmond, has inaugurated a movement to provide a "Queen's nurse" for the sick poor of Newry. The Rev. W. Moore, vicar of St. Patrick's, has consented to be secretary for the time being, and the Lady Superintendent of the Jubilee Nurses' Home, Dublin, will be asked to attend a meeting to advance the project.

### WHAT SOME NURSES WANT.

A short while since a nurse with a month's wages in her pocket in lieu of notice to quit remarked that "her superior officers were not angels." The guardians of Keynsham, in Somerset, could feelingly reply, "No more are nurses." In response to an advertisement for a nurse for the workhouse, a young candidate came to see the place and to be interviewed by the guardians. When, however, the appointment was offered to her she declined it, because there was not a sea view and a tennis court. Moreover, she sent in to the guardians a very substantial bill for expenses, which these unlucky gentlemen allowed because of the expenses involved in contesting it.

### EXTENSION OF THE PLAISTOW MATERNITY CHARITY.

On the 4th inst. a branch home, in connection with the Nurses' Home and Maternity Charity at Plaistow, was opened at East Ham. A house in Plashet Grove has been secured for the accommodation of one trained nurse and three probationers. The short opening ceremony was performed by Mrs. Blomfield, widow of the late Bishop of Colchester, in the absence from sickness of the Countess of Warwick. Afterwards a garden fête was held in the grounds of Durban House, the residence of Mrs. Wilson, hon. secretary, which was largely attended. In the evening it was made still more attractive by a band, illuminations, a concert, and dancing on the lawn.

### A NASTY CHARGE.

All nurses must sympathise with the nurses of the Salford Union Infirmary at Hope, near Manchester, in the very unpleasant incident that took place recently. A patient, who had been suffering from chronic rheumatism for ten years, was taken into the infirmary, which she insisted on leaving three days later. She then accused the nurses of gross violence, declared that they had hurt her back, and knocked a tooth out. It was a nasty charge, especially as the woman died very shortly after her return home. The nurses owe the coroner a vote of thanks for inquiring strictly into the matter, for the fullest evidence was brought forward, and they were completely exonerated. One of the difficulties in dealing with patients of the lower classes is their rooted objection to strict personal cleanliness, and there are hundreds of persons in England who would consider it little short of murder to give an invalid a tepid wash all over. It is easy, therefore, to understand a charge of cruelty being brought against a nurse who performed this very necessary operation. But it is just as easy to see how very important it is that such a charge, however ungrounded, should be publicly refuted.

### HOW AN ENGLISH NURSE FARED IN CUBA.

Some time ago we noted that an English nurse had been expelled from Cuba, and had been taken from the island on H.M.S. Talbot. Now the experiences of Sister Mary Wilberforce are before us. Sister Mary, who is a member of the Royal British Nurses' Association, was induced to go to Cuba by a remark made by the Spanish Ambassador in London as to the need of such services as hers in that place, and she was welcomed on her arrival there by the military authorities, earning their praises for her work. All went well until the exodus of the Americans, which, however, left a community of about 100 persons, chiefly governesses and teachers, practically destitute. Fighting having ceased for the time, and consequently having more leisure, Sister Mary took these waifs under her wing, supporting them out of her own resources, and thereby arousing the jealousy of the Spaniards. To show charity to an enemy was in their eyes unpardonable, and in consequence Sister Mary soon found it impossible to obtain provisions. By good luck some hitch in removing the supplies from the wharves gave her an opportunity of buying what she wanted—an opportunity which she practically lost, because she inserted an advertisement in the daily paper to inform her American friends that she would again distribute medicines and stores from a certain date. The military authorities replied to it by coming and rifling her domain and taking all she had, even to her own private supply of tea. She was then asked to leave Cuba. Sister Mary is now in Jamaica, and intends returning to carry on her work as soon as ever she can.

### PLANS OF THE NEW NURSES' HOME, NORTHAMPTON.

The plans of the new nurses' home at Northampton have been passed by the directors of the Northampton Town and Benefit Building Society, whose approval was necessary, because the building is to be erected on one of their estates. The site has been sold to the joint committees of the Jubilee Celebration and the Nursing Institute by Councillor James Hughes at a very low price. Mr. Hughes has also given them the option of buying the next plot of land. Messrs. Charles Dorman and Son, of Northampton, are the architects. The plans show a three-storeyed building with basement. In the basement are situated the nurses' dining-room, kitchens, &c.; on the ground floor are the matron's room, nurses' sitting-room, office, and store-room; on the first floor are two private bedrooms, the matron's bedroom, a bedroom for three nurses, and a bath-room; on the second storey are cubicles for six nurses, a servants' bedroom, and a bath-room. The estimated cost is £23,00. It is a pity in a new and expensive home not to provide separate bedrooms for the nurses and to place a dining room in a basement. According to the published account of the building the nine nurses are stowed away in six cubicles and one bedroom, an arrangement hardly in keeping with the accommodation now commonly made for nurses in recently erected homes.

### SHORT ITEMS.

Lady Kensington and her daughters, assisted by several distinguished amateurs, gave a concert in the Marloes National School for the funds of the District Nursing Association. The room was crowded by an appreciative audience, and over £5 was taken.—Nurse Holmes, who has been working with the Coventry Nursing Institution, was unanimously appointed district nurse of the parish of Foleshill on July 29th, 1898. —About a hundred members of the British Medical Association visited the Nurses' Home and Maternity Hospital of the Royal Infirmary, Dundee, on the 30th ult.

### Antiseptics for Nurses.

By a Medical Woman.

### XIII.—FORMALDEHYDE.

In quite recent times the antiseptic and disinfectant properties of what is variously known by the names of formaldehyde, formic aldehyde, or formalin have been brought prominently forward, as well as the different antiseptic compounds of which formaldehyde is the base and active principle. At the congress of the Sanitary Institute held last year at Leeds much was said about the wonderful properties of this compound, which has recently been introduced into surgical practice, and which is said to possess antiseptic and disinfectant properties equal to those of perchloride of mercury.

Formaldehyde, or formic aldehyde, is obtained as an oxidation product from the fumes of methyl alcohol, or wood spirit, by passing the vapour of the latter, well mixed with atmospheric air, over red-hot spirals composed of pure platinum. In this way formaldehyde is produced as a white gas, very irritating to both the eyes and the throat. It can be condensed into a liquid and carefully purified from all unconverted methyl alcohol, and freed from any traces of copper or other impurities. It is prepared as an aqueous solution of a strength of 40 per cent., which is the highest point of saturation of the water, which will hold no more, and if it is attempted to make a more concentrated solution a white amorphous substance, called paraform, is obtained. This preparation, containing the highest percentage of formic aldehyde, is known as formalin. Formaldehyde in its gaseous state is readily soluble in water, and forms a clear, transparent, colourless fluid, having a peculiar and very pungent odour, and retaining the irritating effects of the original gas on the eyes and throat. When a solution of formaldehyde is exposed to air at ordinary temperatures it gives off a certain amount of gas and watery vapour for some time, but after a certain proportion of water has evaporated it gradually becomes condensed or polymerised, as it is technically called, and first becomes cloudy, and then a yellowish-white amorphous substance is formed, which is the remainder of the formaldehyde gas, which becomes gaseous very slowly and in very small amounts, and possesses fuller disinfecting powers. Under these circumstances it is best to leave the liquid alone for a while until the cloudy substance has entirely settled, and pour off the clear fluid at the top, which is formalin of full strength, and either add boiling water to the thick part, or warm water and place the vessel in a warm place. Formalin will become thick in a temperature of 80 deg. Fahr. and over, and under 40 deg., but in either case the residue can be treated in the manner described above.

As an aqueous solution of a strength of 1 in 10,000 formalin acts as a very effectual and reliable antiseptic in surgery; it is cheaper than carbolic and possesses equally good germicidal powers. It also possesses the great advantages that it can be used for disinfecting purposes in the form of either a solid, a liquid, or a gas. It is probably in its gaseous form, however, that formalin will be most widely used as a disinfectant, and to facilitate this a special kind of lamp has been devised, known as the Alformant lamp, which has replaced the earlier inventions of Professors Rouse and Baudet. These scientists, finding that all attempts to evolve formalin gas from aqueous solutions of formic aldehyde failed as it invariably becomes concentrated or polymerised when evaporated, undertook a series of experiments which resulted in the discovery that if formic aldehyde gas is dissolved to saturation point in calcium chloride solution, which is then heated, under pressure, in a special apparatus which they devised, consisting of a high pressure boiler with pressure gauge and thermometer, and having a tube and stop-cock for the escape of the formalin gas, and which the originators called an "autoclave," the formalin gas was evolved very satisfactorily and without concentration. The autoclave was placed outside the door of the room to be disinfected, and the tube for the escape of the gas was passed through the keyhole, and when the gauge showed sufficient pressure the stop-cock on the tube was opened and the gas allowed to pass into the room. This method was attended by some inconveniences, and Dr. Merling and Dr. Schmidt, of Berlin, applied the fact that when spirit is burned it produces three parts each of water and of carbonic acid, and used these products to de-polymerise the dry formic aldehyde and convert it into formalin gas by means of the Alformant lamp. The structure of this lamp is extremely simple, consisting merely in a spirit container with burner to be lighted, and above it a pan into which the required number of formalin tablets, according to the size of the room to be disinfected, are placed. The tablets are of a given weight, and consequently the exact amount of gas necessary can be obtained without any trouble. The effect of the heat on the tablets is first to sublime the dry formalin, during which process fumes are given off, and then the gases and water of combustion combine with these fumes and convert them into formalin gas. This method is known as Schering's. It has been found that one tablet or pastille for every 35 cubic feet of space completely disinfects a room, destroying all germs, and even rendering scrapings of dust sterile. In the opinion of competent observers, both in America and on the Continent, who have carried out very careful and thorough experiments with formaldehyde, the gas seems to have solved the problem of completely disinfecting a room and its contents after it has been occupied by a patient suffering from a contagious disease. Formalin gas is, therefore, likely to prove a reliable disinfectant for all surfaces and for such lighter textures as curtains, bed-covers, carpets, clothing, &c., but it must not be depended upon to disinfect articles which consist of several folds, such as bales of materials, mattresses, pillows, &c., nor books. The gas has no injurious effect on wool, cotton, or leather things, nor on fur or silk, and little, if any, change occurred in colour. Of metals, only iron and steel are affected. It is said that when textile goods are subjected to formalin gas much of the gas remains for a long while in the material. This is obviated by exposing such materials to the fumes of ammonia after disinfection with formalin. In disinfecting a room with formalin all openings and crevices must be very carefully closed, as the gas is so diffusible that it is apt to escape without these precautions. When the Alformant lamp, with the proper number of pastilles, has been placed in a room and lighted the room must be left closely sealed up for twenty-four hours. On opening it after the lapse of this time there is a strong odour of formalin, somewhat irritating to throat and eyes, but it is very quickly dissipated if the window is opened, and the room is soon habitable again. Also formalin can be used as a ½ to 1 per cent. solution in water for spraying or washing surfaces, clothes, &c., whilst a 3 per cent. solution is said to render the hands completely aseptic.

An uncovered vessel containing a small amount of formaldehyde can with great benefit be placed in a sick-room, when it will act as a deodorant and keep the air perfectly sweet and fresh, or in cases of infectious disease in a private house sheets soaked in formaldehyde solution and hung up outside the patients' room will diminish the risk of infection. On the Continent a small lamp, arranged to burn very slowly, having one formalin tablet together with either eucalyptus and turpentine mixed, or else eucalyptus alone, has been used in scarlet fever and diphtheria. The odour of the eucalyptus quite disguises that of the formalin. It has also been recommended as an inhalation in cases of pneumonia and tuberculosis.

### Post-Graduate Clinics for Nurses.

By a Trained Nurse.

### ARTIFICIAL METHODS OF FEEDING.—VI.

### Inunctions and Feeding of the Delirious and Insane.

In order to fully complete the subject of artificial feeding, some mention must be made of nourishment by inunction and the forced feeding of patients suffering from mental trouble. Feeding by inunction has proved to be of little value from the nutritive point of view, but there is no question that, employed as it frequently is in cases of marasmus, cancer, or phthisis, it improves the general condition of the patient. Such manner of feeding is rarely used for adults, being confined chiefly to children and infants, in whom it produces benefit rather from the advantage gained by the massage and friction which accompanies this form of feeding than from the amount of nourishment which is actually absorbed from the skin.

Olive oil, and in some cases cod-liver oil, is employed, the nurse putting from half to one teaspoonful of the warmed oil in her hand and rubbing it in gently over the thighs and abdomen. She must carefully avoid hard rubbing since this would be likely to set up too much intestinal activity, and in the marasmic and phthisical infant a diarrhœal tendency frequently exists, or may easily be set up by abdominal friction. The inunctions may be repeated two or three times during the twenty-four hours, cacao butter being preferred by some physicians. Practical experience leads one to the view that, even though inunctions may be of little value in themselves, they are valuable in nourishing and softening the harsh dry skin of wasted infants, and that by improving the blood circulation the patient is enabled to digest and assimilate more food. Inunctions assuredly increase the appetite, and, by improving the tone of the intestinal tract the patient seems to gain a distinct access of assimilative power, and the children for whom inunctions are ordered are usually such wasted cold little mortals that the physical well-being, warmth, and comfort produced by the rubbing well repay the trouble of the process. The peevish wasted infant usually passes into a comfortable sleep, especially if the inunction has been performed, as it should be, before a fire, so that hygienically if not dietetically inunctions are valuable. One practical point of which the nurse may be reminded is that when patients are being fed by enemata, or in such artificial fashion as to limit the amount of nourishment taken, their rooms require to be kept specially warm. A temperature of 68 deg. to 70 deg. F. is by no means too high for a patient depending on enemata for his sustenance. For it must not be forgotten that the first demand made by the body upon the food taken is made for the purpose of keeping up vital heat, so that the colder the patient is the more nourishment will be absorbed for the mere purpose of keeping his body warm, and there will be less residue to be applied to general nutrition. Hot bottles and warm room are therefore essential to the patient fed by nutrient enemata, especially as heavy bed-coverings are not permissible for patients suffering from the exhaustion produced by such insufficient feeding. It is important, too, to save the strength in every possible way. The patient must do nothing for himself, and the nurse must husband with miserly care the small strength remaining to a sick person who relies on rectal injections for his food source. A flannel abdominal binder is helpful in such cases, as it keeps up a uniform temperature in the intestines, and in this way and by preventing chills puts the artificial digestion under the best possible conditions. Well nourished and stout patients generally do better on nutrient enemata than do the thin and spare, since the former draw upon their own reserve store of nutriment.

I omitted to mention in my list of the cases for which nutrient enemata might have to be employed, those cases of persistent vomiting in pregnancy when the patient is much reduced by constant sickness. When the vomiting proves insurmountable, some medical men treat the case by permitting nothing by mouth for some days, relying entirely on injections. In extreme cases of sea-sickness, too, this plan is sometimes adopted with great benefit to the patient. For the stomach "gets into the habit" of vomiting, and such a habit is easiest broken by rectal feeding.

In conclusion of the subject, a few words must be said as to the feeding of patients suffering from delusions, melancholia, or any form of mental or nervous trouble which leads to the refusal of food. In mental cases—or in the delirium of fever—curious prejudices and hallucinations with regard to food arise temporarily. Such patients may refuse food from one person and take it readily from another. In such conditions the nurse must do her best to discover the idea underlying the prejudice. The prejudice may originate in a morbid belief that poison is added to the food. It sometimes happens that such a patient will take nourishment by himself when he will not take it from his nurse. She should then place it by his bedside, busy herself at the opposite end of the room, and ignore the fact that the food has disappeared.

In mania and melancholia a patient may sometimes be cured of his refusal of food by being fed by the stomach-tube, the nurse tactfully pointing out that swallowing the food in the normal manner is a much more pleasant and easy method of taking meals. It is a very difficult matter to feed a resisting patient through the œsophageal tube, since he may absolutely refuse to swallow it. The nasal tube presents less difficulty in violent cases, but it is as well to encourage the patient to talk whilst this is slipped into position, so that if it should by chance pass into the larynx the fact will be instantly discovered. Nutrient enemata may prove of little value in mental and melancholic cases, for such patients quickly discover the purpose of the injections and learn how easily these may be rejected.

In the feeding of mental cases—forced or otherwise—care must be taken that the diet selected be easily digested, for emotion and excitement have a marked effect on digestion. Added to this is the fact that in cases complicated by brain trouble, temporary or permanent, the patient is usually very careless about mastication and "bolts" his food almost whole. Where feeding by tube is employed, in cases complicated by brain affections, in suicidal melancholia and nervous depression, very large quantities must be administered at each meal, for forced tube-feeding is not a pleasant or easy process. A quart of milk thickened with some malted food and three or four whipped eggs may be given twice or three times in the twenty-four hours. The nurse must invariably bear in mind that the absence of the action of the saliva on tube-swallowed food necessitates the addition in all such cases of malted meal or malt extract.

One of the most trying points in the feeding of patients delirious or violent rests in the fact that nothing quiets their condition so much as effectual and constant feeding, and their refusal to take just the one essential to their welfare is very distressing. In such cases much undoubtedly rests with the nurse, who may by tactful patience induce the most refractory persons to take food. Once having found out the particular foods such patients most like, the nurse may sometimes tempt them through their own inclinations. But I cannot promise success in this direction to the most skilled nurses, nor can I promise that the artificial feeding of insane and delirious patients will ever assume a pleasant aspect. But the good nurse will never lose heart or patience in these trying cases, but will go on with the task of devising new expedients for feeding and keeping up the strength of the most hopeless and most wearying examples of sick cases that a nurse will be called upon to care for.

### A Letter from Bombay.

By a Sister on Duty.

### NURSING THE PLAGUE AT KARACHEE.

Since I last wrote about my "plague" experiences in Bombay I have spent a little over two months nursing in Karachee, where the Civil Hospital had been converted into "plague" wards; the patients suffering from other illnesses having been removed to a large college then in disuse.

We had a nursing staff of five English sisters, two Indian nurses, and also a native staff of ayahs and ward boys. It is quite a pleasure sometimes to teach these native assistants, who are often very intelligent. I remember one ayah in particular, who, after a few weeks' training in the wards, grew remarkably clever. It was quite pleasant to see the capable way in which she would prepare a patient for examination, or to watch her getting things ready for a dressing, the deft way in which she placed the mackintosh under the limb, removed the bandage, and had everything ready in the way of a table, carbolic, receiver, dressing box, &c., and to see her delight if she were allowed to do some simple dressing under my supervision. I have often thought since then what a capital nurse she would have made, and it seems such a pity that she, only a poor Hindoo widow, should lack the opportunity of learning to excel.

Karachee is a seaport town, and it is two days' journey by sea from Bombay. This I shall not describe, as I still have a lively recollection of the horrors of that voyage. Our first impression of Karachee is not pleasant; it appears to be nothing but a sandy waste, with little to relieve the monotony of the scenery, as all the houses seem to be built of shady-coloured brick; but on nearer acquaintance it is very interesting, and the number of camels give it quite a picturesque and Oriental appearance. By-the-bye, I may here add I once went for a ride on a camel, and I shall never forget it. The saddle is placed sideways, and to enable one to mount the camel has, of course, to lie down, and I really do not know which is worse—the down-lying or the uprising. I only know I was first conscious of four great upheavals, which appeared an eternity, after which I realised it was at last on its feet, and then began a series of jerks, during which I found it very difficult to keep my hat on. It is impos ible to describe the motion of a camel while it continues its uneven course; I only know I was not sorry when the camel, after four huge plunges, lay down to enable me to dismount. However, I enjoyed the novel experience.

The "plague" in Karachee was worse than in any other town in India, and the death-rate was very high. We also noticed that the buboes assumed a different aspect and appeared a great deal worse than any we had seen either in Bombay or Poona. In some cases they formed huge cavities which took a long time to fill in.

The hospital was built of stone, which we found very nice in the hot weather, and everything that was possible was done for the care and comfort of our patients. Fruit was supplied daily for those who could take it, and we had the best of brandy, port wine, Brand's essence, &c., for our bad cases. Dolls and toys were provided for the children, and, indeed, in many cases the young married women were just as keen on having them as the little children, and I have often been amused to see the young mother of two children sleeping contentedly with a doll in her arms. Most of these native village women had never seen English toys, and were delighted with the golden-haired dolls.

After a few weeks of hard work the "plague" began rapidly to decrease, and when we left last week for Bombay we only had twelve convalescent cases in the wards, under the care of two nurses, though, of course, a few cases daily are still discovered; this is a vast contrast to just a few weeks before, when not only every ward in the hospital was quite full, but temporary sheds had to be erected all over the grounds in order to admit the cases which seemed to be coming in ceaselessly.

The climate of Karachee is wonderfully good, and we only experienced a few very hot days; there is nearly always a cool breeze blowing, and provided we kept our doors and windows open, it was very nice, but on the few occasions when there was no breeze, it was very hot. I cannot close my letter about Karachee without mentioning what a happy time we spent there. We are much indebted to Surgeon Lieut.-Colonel McCloghery for all his kindness to us, for he helped us in every way, and I shall also never forget the kindness and hospitality we received from everyone. Ladies used to come for us in the evenings and take us out for charming drives where we could benefit by the sea breezes, which we thoroughly enjoyed after our day's work. We also went to several very enjoyable "At homes" and dinner parties, and indeed nothing could have exceeded the kindness we received. Needless to say, we were very sorry to leave.

After another two days' very rough sea journey we arrived last week in Bombay, where I must say the climate is very trying. We are in the midst of the Monsoon, which means days of ceaseless rain, during which time all our clothes, boots, gloves, &c., take the opportunity of getting spoilt. A general feeling of dampness pervades the air, and the heat is very trying.

At present there are several nurses "off" duty, as "plague" has decreased everywhere, and there have been twenty-three of us staying at the Great Western Hotel, as nearly all the "plague" hospitals are closed. At first we were rather alarmed at the thought of the expense of an hotel, but as Government has arranged to pay three rupees a day for each nurse, and we have only to pay one rupee each, it is, on the contrary, decidedly economical. It is quite a novel experience for us to be doing nothing, and I think we are all glad of the rest during this hot weather. There are rumours that we may expect a fresh outbreak directly the cold weather begins, and preparations have already been completed in Calcutta. Some of the nurses have been sent there, but from all accounts we are not anxious to join them, as it seems to be very expensive, and last week the new rules appeared in the *Bombay Gazette*, on which it is difficult to offer any comment (see "Mirror," August 6th). I can only say that it has not been necessary to enforce such rules in any hospital where I have worked before, either in Poona, Bombay, or Karachee, and until we hear further particulars it is impossible to understand them, besides which our income would be quite inadequate for the expense of living in Calcutta. However, I hope to have further details later on, for we have always found hitherto that the expenses of the sisters on "plague" duty have been carefully considered.

### Presentations.

On Tuesday, August 9th, Miss Wand, for the last fourteen years nurse-matron of the Westminster Memorial Cottage Hospital, Shaftesbury, was presented with a cheque for £50 17s. upon her leaving the hospital. Sir R. G. Glyn, in making the presentation on behalf of numerous friends and old patients, expressed the general regret at her resignation, which, owing to her lameness, she had been obliged to send in. Miss Wand's services to the hospital have always been appreciated by her committee, and by both medical officers and patients. Her successor is Miss L. H. Harris.

### Everybody's Opinion.

[Correspondence on all subjects is invited, but we cannot in any way be responsible for the opinions expressed by our correspondents. No communication can be entertained if the name and address of the correspondent is not given, as a guarantee of good faith but not necessarily for publication, or unless one side of the paper only is written on.]

### LADIES v. GENTLEWOMEN.

"A Nurse" writes: Will you allow me space in your valuable paper to reply to "Matron's" letter of last week, in which she writes complaining of the number of unsuitable applications she receives in answer to her advertisements for gentlewomen as probationers. I do not agree with "Matron" that the term gentlewoman necessarily implies good birth and education. My definition of gentlewomen is women of refined and gentle natures, whose minds are pure and holy, no matter whether they be rich or poor, high born or low born, educated or uneducated. So that if "Matron" requires the addition of high birth and college education she should state it in her advertisement. If she did so not only would she save herself trouble and spare the poor girls anxious to become nurses keen disappointment, but she would save the hospital where she is employed unnecessary expense of postage, because none but women who could boast of high birth and college education would answer her advertisement. Now, as "Matron" says, she feels in honour bound to write to those unfortunate applicants, and consequently she spends a great deal in postage.

### NURSING THE PLAGUE.

"One of the Lady Nurses" writes: May I call your attention to an extract in the *Indian Daily News*, of to-day's date, a copy of which I am sending to you by this mail I think it would be well if you would give publicity in The Hospital to the rules which have been made for the lady nurses engaged on plague duty in Calcutta, for they are of a most obnoxious nature, and such as never ought to have been imposed upon volunteer nurses; but, bad as they are, they are trifles compared with the constant bye-rules and regulations of which they are made the basis. The uses of discretionary power by the lady doctor does not work well, and life is made a daily, nay, hourly burden to us. I think it is only fair that possible volunteers should have an idea of what they may be subjected to if they are sent out to Calcutta on plague duty—gentlewomen, trained nurses, and women of from twenty-eight to thirty-five years of age, not as one would gather from the rules, women of the lower classes, or school girls just let loose on the world. I append my name and address as a guarantee of good faith, and earnestly request you to give publicity to this letter, and the rules and comments in the *Indian Daily News*.

A Medical Correspondent writes: In referring to the rules relating to nursing of the plague in last week's Hospital, it is only fair to say that it is only in Calcutta that such indignities have been offered. In other places they have been fairly provided for, but while the plague was raging work was arduous and unpleasant. It has pleased Government to issue a resolution recently that those sisters who wish will be permitted to renew their engagement for the period of another year, but no extra salary is offered to them and Government may dispense with their services at a month's notice after the first extra six months' appointment. All the nurses will be only too glad to return home as soon as they can get released from their present engagement. The pay of R175 and quarters is not very attractive as expenses are heavy and discomforts and annoyances numerous. Just now, while there is a lull in the virulence of plague in Bombay, many of the sisters are idle, but unfortunately there are indications of the rapid development of a recrudescence. Should plague increase and Government apply for more nurses I cannot advise well-trained English women to come out under the existing conditions. They should have more pay, and they should be free from the possibility of being subjected to the school-like regulations adopted in Calcutta. A lady doctor is there, acting as matron to the establishment, and the regulations made at one of the hospitals are described as still more exacting and vexatious. The method adopted by the Indian medical authorities against anyone who displays any objections to such rules is apparently to send them away to some out-of-the-way place, where life is simply an imprisonment. Such a course may be meted out to refractory members of the semi-military medical service, but it is disgraceful young English women, fresh from home, should be subjected to such treatment. Did the authorities at home know of this, I feel sure it would be quickly remedied.

### A Sister's Lament.

I like to teach probationers,
But I far too often find
That they're possessed of memories
Of a very moderate kind.

Duties are sometimes overlooked,
Reports are incomplete,
And the way in which the work is done
Is anything but neat.

On the charts the dots and figures
Are of wondrous shape and size,
And if by chance a line is straight
I can't believe my eyes.

And then this sort of thing I find
When looking at the slate,
They forget to give the milk that's due
Before they go at eight.

Then for changing fomentations
Alas! the water's never hot;
It wants but little forethought,
And that they haven't got.

To drill them into doing things
In just the proper way
Takes such a lot of hammering,
It nearly turns me grey.

Still success should crown my efforts,
If they're made of proper stuff,
For they say "All comes to those who wait,"
If they wait but long enough.

### The Grosvenor Hospital for Women and Children.

We have received a letter from one of the oldest governors of the Grosvenor Hospital for Women and Children fully corroborating the appreciation of Miss Hughes' life and services to the hospital which appeared in our issue of last week. Our correspondent points out that the hospital is fortunate in still possessing many other old and valued officers who have served it for long periods. We are glad to be able to give the following additional particulars of Miss Katherine Hughes' training. She received a three years' course at the University College Hospital, and at the end of that time she was appointed lady superintendent of St. Patrick Dunn's Hospital, Dublin, which post she only held a few months. After leaving it she was appointed lady superintendent of the Grosvenor Hospital, which post she has held over twenty years. But she never worked at the London Hospital as was stated in our former notice from information received from the Grosvenor Hospital authorities.

### Appointments.

### MATRONS.

Montgomeryshire Infirmary, Newtown, North Wales.—Miss Fanny A. Colman has been appointed Matron of the above infirmary. She was trained, and subsequently staff nurse and sister at the Royal Hants County Hospital, Winchester. She then became charge nurse at the Fountain Fever Hospital, Tooting, and afterwards was night superintendent of the hospital ships, Dartford, and then at the Grove Farm Fever Hospital. She is a member of the Royal British Nurses' Association, holds the L.O.S. certificate, and that of the Apothecaries' Hall for dispensing.

Manchester Royal Eye Hospital.—On August 3rd, 1898, Miss M. E. Bland was appointed Matron of this hospital. She was trained at Sheffield General Infirmary, and was matron of Beverley Hospital and Dispensary from September, 1893, to May, 1896, and she is at present lady superintendent of the Hull Municipal Hospital for Infectious Diseases.

### The Book World for Women and Nurses.

[We invite Correspondence, Criticism, Enquiries, and Notes on Books likely to interest Women and Nurses. Address, Editor, The Hospital (Nurses' Book World), 28 & 29, Southampton Street, Strand, London, W.C.]

"Perish the Baubles." By Frances H Wood. (London: Simpkin, Marshall, and Co. 1898.)

Miss Wood's little story is full of exciting incidents and thrilling adventures, and will commend itself to those to whom the sensational is dear. It is a story about a weird old country house with secret chambers and passages, which is the scene of a daring burglary of the celebrated Moberly diamonds. At one stage in this book, when the excitement of the robbery is at its height, the reader can barely see how the ending can be anything but a tragedy. Eventually, however, matters right themselves, and all winds up peacefully and happily. Miss Wood's little volume is neatly and effectively got up at the modest price of 1s., and it is an entertaining bit of reading for the leisure hour.

A Manual of Massage. By M. A. Ellison, L.O.S. (Baillière, Tindall, and Cox. Price 3s. 6d. net., 124 pages and 50 illustrations.)

When Miss Ellison comes to the practical massage in her manual she gives some admirable professional hints, and writes as an expert. It is the greater pity, therefore, that more than half her book is devoted to anatomy, which has been much better done in many other books. The masseuse, after learning her profession practically, for massage cannot be learnt from books, wants a manual of her art, and not a handbook of anatomy. If Miss Ellison were to leave the anatomy to others, and were to enlarge on the art of massage, her book would be really valuable to nurse and professional masseuse alike. When Miss Ellison writes of massage it is evident that her knowledge is very thorough, and because that part of her book which is devoted to massage is so good one regrets that so much space has been taken up by a mere description of bones. For anatomy, like massage, must be learnt practically, and not entirely from books. In the section on Weir-Mitchell treatment no word appears to be said of the inevitable daily sponge bath, without which the famous specialist would certainly not allow massage, under any circumstances, to be performed on any patient. A daily sponge-bath is essential to a patient undergoing massage. Directions are also given to "continue the massage of the limbs" during menstruation. This is not "Weir-Mitchell treatment" as carried out by the Philadelphian (not New York as stated in the book) physician. Neither would he choose—as Miss Ellison prescribes—a nurse with "plenty of small talk." The silence of a nurse in rest cases is indeed golden. Miss Ellison, with her knowledge and experience, could write a much better book than she has achieved, although, with a few minor exceptions, the massage section of the volume before us is admirable.

The History of Carlisle Cathedral. By R. F. Ferguson. (Isbister and Co.)

Messrs. Isbister and Co. have issued another dainty little volume of our English cathedrals, forming one of a series to which we have already referred at greater length in these pages. "The History of Carlisle Cathedral," by R. F. Ferguson, Chancellor of Carlisle, is the latest addition, and this book maintains the high standard of excellence set by its predecessors in the series. Carlisle Cathedral, as most of us know, consisted of two churches, that is to say, there were two churches within one building. The original church—Athelwold's church—was a Norman minster of the twelfth century of moderate size, of which there are still fragments to be seen. The bays to the west were destroyed by fire in 1646, and a quaint entry in the Library of Lambeth in a survey of the year 1649 runs: "It appears onto us by ye view of judicious workmen that ye decayes of the church of S. Maryes of Carlyle is such that ye weste pte being taken down the repair of the east wtl coste fyve hundred pounds att ye least whch seems to us very necessarye their beeinge but one other Church (wch is likewise very Ruynas in all that Citty)." The necessary funds for its rebuilding not being forthcoming, the parish church was then amalgamated with the two remaining bays of the nave.

### The Milkmaid Nurse: An Irish Jumble.

The qualifications which are required in those who would spend their days in tending the helpless and nursing the sick are increasing in number every year. It is no longer possible for those who feel an inclination or an aptitude straightway to enter on the duties of a nurse. Anatomy and physiology and many other dreary details have to be mastered before one is allowed to pose as the ministering angel or to soothe the aching brow. It has been left, however, to the guardians of the Coleraine Union to give the final touch to the nurses' curriculum, and to add still one branch of culture to the wide range of subjects on which those who tend the sick are expected to be skilful. They want a "strong healthy woman to act as attendant for the female infirmary and lunatic wards." Not a word is said as to her training, her knowledge of infirmary nursing, or her experience in dealing with lunatics. The only qualification is that in addition to being strong and healthy "preference will be given to one who has been accustomed to milk cows!" Well done, Coleraine! We have had many articles lately upon the details of milk diet and on the preparation of milk foods, but with all our desire for thoroughness we confess it never occurred to us to go quite so far back to the beginnings of things as Coleraine has done, or to demand that a nurse must begin by being a milkmaid! But, seriously, what are we to think of a board of guardians who, when seeking for an attendant on the sick, the helpless, and the insane, instead of demanding some experience, some knowledge, or some training in the work she will have to do, demands only that the woman appointed to this important office shall be able to milk cows? Those who can read between the lines know well enough the abominable nature of the whole device. Boards of guardians are now compelled by law to do away with pauper nursing, but nurses they do not love. It is not only the wages they have to pay that they dislike; too often it is the cleanly, tidy ways which nurses introduce and the higher type of attendance on the sick that they enforce which go against the grain with the lower-class guardians. Hence these attempts, which we see on every side, to introduce what we may call general utility women instead of nurses. Those who think that nursing consists in straightening the bedclothes when the doctor comes round cannot see what nurses find to do, so they set them to scrub or to sew or to bathe the casuals or to make puddings, or even, as a last stroke of genius, to milk cows. But all this is sorry work, and shows on the one hand a great indifference to the wants of the poor who have come under their guardianship, and, on the other, a most improper desire to escape by mean subterfuges from the duty laid on them by law of providing efficient nursing for the sick.

### Minor Appointments.

The Eversfield Hospital, West Hill Road, St. Leonards-on-Sea.—Miss Lucy E. Aldridge has been appointed Secretary and Dispenser to this hospital. She holds two certificates of proficiency—one from the Apothecaries' Hall to act as an assistant in compounding and dispensing medicine; the other from the Westminster College of Chemistry and Pharmacy. Miss Aldridge is also a trained nurse, having received her instruction at the London Hospital.

### For Reading to the Sick.

### THE LIGHT OF THE WORLD.

**Verses.**

Father, I know that all my life
Is portioned out for me,
And the changes that are sure to come
I do not fear to see;
But I ask Thee for a present mind
Content on serving Thee.

I would not have the restless will
That hurries to and fro;
Seeking for some great thing to do,
Or secret thing to know;
I would be treated as a child,
And guided where to go.

There are snares besetting every path,
That call for patient care;
There is a Cross in every lot,
And an earnest need for prayer;
But a lowly heart that leans on Thee
Is happy everywhere. —*E. W.*

Oh, blessed Lord! How much I need
Thy Light to guide me on my way;
So many hands that, without heed,
Still touch Thy wounds and make them bleed;
So many feet that day by day
Still wander from Thy fold astray;
Feeble at best is my endeavour!
I see but cannot reach the height
That lies for ever in the Light;
And yet for ever and for ever,
When seeming just within my grasp,
I feel my feeble hands unclasp,
And sink discouraged into night—
For Thine own purpose Thou has sent
The strife and the discouragement. —*Longfellow.*

Lead, kindly Light, amid the encircling gloom
Lead Thou me on;
The night is dark, and I am far from home,
Lead Thou me on.
Keep Thou my feet, I do not ask to see
The distant scene, one step enough for me.
—*Newman.*

**Reading.**

"I am the light of the world."—*St. John* ix. 5.

The sun ariseth and man goeth to his work until the evening. It is thus with the sun of righteousness. When He riseth, there is light in the Church, light in the house, light in the cloudy day, and light in the dark valley.

1. The calls to repentance, the preaching of the Lamb of God, the in-dwelling of the Spirit, sound doctrine, holy sacraments, godly discipline, good works, cross bearing, growth, a missionary spirit, an open Bible, a form of sound words—that is "Light in the Church."

2. Mutual love, prayers unhindered, children brought to Jesus, the household ordered in the fear of God, the family altar reared, the holy church frequented, the Lord's Day hallowed, God's ministers honoured—that is "Light in the house."

3. Sickness endured patiently, reproach borne meekly, injuries forgiven readily, disappointments taken cheerfully, bereavements suffered unrepiningly, the future looked to hopefully, Christ sought lovingly—that is "Light in the cloudy day."

4. The soul at peace, death disarmed, the conscience void of offence, love driving out fear, the look upwards, "the heavens opened and the Son of Man seen standing on the right hand of God"—that is "Light in the dark valley."—*J. B.*

### Notes and Queries.

**The contents of the Editor's Letter-box have now reached such unwieldy proportions that it has become necessary to establish a hard and fast rule regarding Answers to Correspondents. In future, all questions requiring replies will continue to be answered in this column without any fee. If an answer is required by letter, a fee of half-a-crown must be enclosed with the note containing the enquiry. We are always pleased to help our numerous correspondents to the fullest extent, and we can trust them to sympathise in the overwhelming amount of writing which makes the new rules a necessity.**

**Every communication must be accompanied by the writer's name and address, otherwise it will receive no attention.**

*Well Qualified.*

(181) I should like to know if the combined three-year certificate offered by the Victoria Park Hospital to probationers having had two years' satisfactory training at that hospital and one year's training at a general hospital is equal to that granted for three years' service in a general hospital. Would I have any difficulty in obtaining work in a good hospital afterwards?—*M. A. G.*

The combined three-year certificate offered by the Victoria Park Hospital has been accepted as qualifying its holder for registration by the Royal British Nurses' Association, and any nurse possessing it would be eligible, not only for work in good hospitals, but for good positions in most hospitals.

*A Nursing Home.*

(182) Can you tell me of a nursing home where a lady of small means, suffering from internal cancer, could be received? She is living in the Isle of Wight, and would prefer remaining there if possible. I should be glad of the names of nursing homes elsewhere where chronic cases are received.—*Enquirer.*

Have you consulted "Burdett's Hospitals and Charities" (Scientific Press, London)? You will find in it a list of all such homes, with particulars. St. Saviour's Hospital, Osnaburgh Street, Regent's Park, is very comfortable.

*"The Lady's Residences."*

(183) Can you tell me the address of an institution in or near Sloane Street where private nurses can get rooms? I believe there is a restaurant in connection with it, and that it is called "The Lady's Residences."—*Nurse Margaret.*

Apply 52a, Sloane Street.

*Marylebone Infirmary.*

(184) Could the Editor kindly tell me if Marylebone Infirmary is considered a good recognised training school for nurses, and would it be advisable to be trained there in preference to a general hospital with 100 beds?—*F. Ki lett.*

The fact that the Marylebone Infirmary is one of the places to which probationers of the Nightingale School are sent for their training, is a sufficient recommendation.

*Dandruff.*

(185) I should be most grateful if you could tell me anything that would cure dandruff, and prevent most unpleasant irritation of the head. Sometimes the skin comes off almost as if it were peeling. I should also be pleased to know if there is a book published on bandaging.—*Honor.*

From your description you ought to be under medical treatment. The irritation may be relieved by washing the head in soft water (artificially softened if rain water cannot be procured) and a bland soap about once a week, and using a hair wash every day. Borax and camphor is good and inexpensive.

*Home Nurse.*

(186) (1) To whom must nurses apply for information respecting the new Schol Board nurses of whom you have spoken in your columns? 2) How to set about getting patients who only require a nurse's services for a short time in each day? I am very anxious to get some work in London in which I could have rooms of my own, so that I may have a chance to make a little home for an invalided brother, and I am not strong enough for ordinary district work in London.—*Hiraeth.*

(1) Miss Honnor Morten, Ivy Hall, Richmond, is the hon. secretary of the London School Nurses' Society. (2) Miss Wood, the Nurses' Hostel, Francis Street, N.W., supplies visiting nurses from her private staff. She would perhaps kindly advise you. You must make sure of a connection before you make your venture.

*The Nauheim Treatment.*

(187) I should be glad of particulars as to the Nauheim treatment for the heart. Can English nurses be trained there; if so, at about what cost? Can you tell me how long the training takes, and if the season lasts all the year?—*E. Forlonge.*

The principal season at Nauheim is from May to October; but we believe that a certain number of patients resort to Nauheim at all times of the year. We do not know of any special arrangements for the training of English nurses at Nauheim, but German-speaking Englishwomen would be sure to find opportunities, as others do, for obtaining training in the work that is done there. So far, however, as regards the "exercises," they are now taught in England at the various schools of massage.

*Home Hospital Charges.*

(188) Please tell me what would be the average charge in a private house for an ordinary medical or surgical case. I am opening a new house for boarders, and one of the local doctors has asked me to keep one of the best rooms for a sick room.—*Policy No.* 443.

You must reckon your charge by your expenses. First find the value of the room, rent, food, attendance, nursing, and the profit you must have to make it pay you, and then never charge less. From your description £2 2s. would appear sufficient to cover the expenses of an ordinary boarder, and you must add to that nurses' fees and keep, sick diet, &c.

